# Self-reported impact of caregiving on voluntary home-based caregivers in Mutale Municipality, South Africa

**DOI:** 10.4102/phcfm.v8i2.976

**Published:** 2016-05-31

**Authors:** Ntsieni S. Mashau, Vhonani O. Netshandama, Makondelela J. Mudau

**Affiliations:** 1 Department of Public Health, University of Venda, South Africa; 2 Community Engagement, University of Venda, South Africa; 3 University Income Generation Center, University of Venda, South Africa

## Abstract

**Background:**

The establishment of home-based care (HBC) programmes in developing countries has resulted in a shift of burden from hospitals to communities where palliative care is provided by voluntary home-based caregivers.

**Aim:**

The study investigated the impact of caregiving on voluntary home-based caregivers.

**Setting:**

The study was conducted at HBC organisations located in Mutale Municipality of Limpopo Province, South Africa.

**Methods:**

A quantitative cross-sectional descriptive survey design was applied to investigate the impact of caregiving on voluntary home-based caregivers. The sample was comprised of (*N* = 190) home-based caregivers. Home-based caregivers provide care to people in need of care in their homes, such as orphans, the elderly and those suffering from chronic illnesses such as tuberculosis, HIV and/or AIDS, cancer and stroke. Self-administered questionnaires were used to collect data which were analysed descriptively using the Statistical Package for the Social Sciences software, Version 20.

**Results:**

The results showed that 101 (53.2%) participants were worried about their financial security because they were not registered as workers, whilst 74 (39.0%) participants were always worried about getting infection from their clients because they often do not have protective equipment.

**Conclusion:**

Voluntary home-based caregivers have an important role in the provision of palliative care to people in their own homes, and therefore, the negative caregiving impact on the lives of caregivers may compromise the provision of quality palliative care.

## Introduction

Home-based care (HBC) programmes throughout the world were found to be effective in easing healthcare institutions such as hospitals of overcrowding, and people who were suffering from chronic illnesses were cared for at home by family members. In the United States, ‘most elderly people prefer to be cared for at home even though there is no infrastructure to support the provision of high quality home-based care’.^1^ HBC in the United States is also referred to ‘as informal care because it is provided by relatives, friends and neighbours’.^2^ The ‘burden of care on home-based caregivers is increased by the fact that home-based caregivers come across different problems when caring for homebound seniors with several chronic medical conditions in the home environment’.^1^ Home-based caregivers in the United States experienced ‘physical, emotional and economic pressure resulting from long-term caregiving demands, which even lead to burnout of people caring for chronically ill home-bound individuals’.^2^ The establishment of HBC programmes in developing countries such as South Africa has ‘resulted in a shift of burden from hospitals to the community where palliative care is provided by voluntary home-based caregivers’.^3^ In South Africa:

home-based care programmes are directed at healthy people, risk or frail older persons, risk people with moderate to severe functional disabilities, people recovering from illness who are in need of assistance, terminally ill persons, persons living with HIV/AIDS or any other chronic disease and any other disadvantaged group/person in need of care as stipulated in the national guidelines.^4^

According to the World Health Organization,^5^ ‘most of home-based care (HBC) organisations are developed as volunteer programmes and registered as Non-Profit Organisations (NPOs)’. The result is that the services of HBC organisations become:

overcrowded and depressed by the number of patients since home-based caregivers are responsible for the provision of care to people in need of care in their own homes such as those suffering from HIV/AIDS, tuberculosis, cancer and other chronic diseases which require palliative care.^6^

Because the increase in the number of orphans, voluntary HBC work has extended to the care of orphans and the elderly.^7^ HBC work was found to be demanding and stressful for the voluntary home-based caregivers.^5,6,7^ Despite the development of HBC guidelines and legislations, voluntary home-based caregivers find themselves performing other roles such as household chores, including cooking.^8^ Hence, the study was aimed at investigating the self-reported impact of caregiving on voluntary home-based caregivers. The study objectives were thus set to determine the self-reported impact of caregiving on the physical, mental and financial well-being of voluntary home-based caregivers.

## Research methods

### Study design

A quantitative approach was followed, using a cross-sectional descriptive survey to investigate the self-reported impact of caregiving on voluntary home-based caregivers. The design was preferred for this study as it allowed the probability of including all voluntary home-based caregivers in Mutale Municipality and therefore avoiding bias.

### Setting

The study was conducted at Mutale Municipality, which is situated in the far North Eastern part of Vhembe District. Vhembe District is one of the five districts of Limpopo Province in the Republic of South Africa. Mutale Municipality comprises about 24 239 households spread in 150 villages under the leadership of traditional leaders such as chiefs and headmen. Mutale local municipality had no hospital; instead, the community depended on hospitals which were located in other municipalities. There was only one health centre and 17 primary healthcare clinics. There were about 26 HBC organisations that were registered with the Department of Health and Social Development and only 4 were not receiving funding from the Department of Health and Social Development.

### Study population and sampling

The population comprised a total of 390 voluntary home-based caregivers working in 26 HBC organisations in Mutale Municipality. All 26 HBC organisations in Mutale Municipality were included in the study. Based on the sampling frame of 390, sample size of *n* = 197 was calculated using Slovin’s formula (*n* = *N*/{1 + *Ne*^2^}), where *n* denotes the sample size and *N* population size with *e* = 0.05 as the margin of error.^9^ Simple random sampling was used to select participants from each HBC organisation. The sample size was proportionally divided according to the population of each HBC organisation to ensure proportional representation of participants in the final sample.

### Instrument and data collection procedure

A structured questionnaire was designed to obtain appropriate information regarding the impact of HBC from voluntary home-based caregivers. The questionnaire was developed in English and translated into Tshivenda by a language expert from the University of Venda, and it was then translated into English by another language expert to ensure the consistency of the instrument. A wide range of literature was used in the development of the instrument, and the objectives of the study were considered. The questionnaire comprised one section on biography and another section on the impact of caregiving. Participants were informed about the purpose of the study before the questionnaire was administered.

Validity and reliability of the instrument were established in order to ensure the quality of the results. Face, content and construct validity were ensured during the development of the instrument to ensure that the instrument measures the impact of caregiving on voluntary home-based caregivers. The instrument was thoroughly scrutinised by the supervisors of the study and senior members of the University of Venda Higher Degree Committee who gave comments. Comments were used to rephrase and reconstruct the instrument so as to suit the level of understanding of the participants. A test–re-test was done to check the stability and reliability of the instrument on 10 volunteers in a HBC organisation with similar characteristics to the target population by re-administering the questionnaire after 1 week. The results showed high reliability with a correlation coefficient (*r*) of 0.99.

Data were collected after permission to conduct the study was obtained from the Limpopo Province Department of Health and Social Development, Vhembe District and Mutale Municipality. Arrangements were made with the managers of the HBC organisations around Mutale Municipality on the dates for data collection. On the day of data collection, HBC offices were used with the permission of the HBC manager. Participants were informed of their rights to withdraw from completing the questionnaire if they felt like doing so and were also advised not to write their names on the questionnaire. Two trained research assistants administered the questionnaire to participants after written informed consent was obtained. Participants were all Tshivenda speaking, same as the researchers, and the Tshivenda version of the instrument was used. It was therefore possible to communicate with the participants in the language they understood. The researcher and the research assistants were available during the completion of the questionnaire to assist participants in case they experienced difficulties in the completion of a questionnaire or in case they needed clarity. It took about 1 hour for the participants to complete the questionnaires.

Participants who could not read and write were assisted by the researcher and the research assistants in completing the questionnaire. Questionnaires were completed in the presence of researchers and were collected immediately after completion. Of 197 questionnaires that were administered, 190 were returned.

### Data analysis

Data were coded and analysed using Statistical Package for the Social Sciences, Version 20.0 software. Descriptive statistics were used because they provide meaningful summaries about the variables.

### Ethical consideration

Approval to conduct the study was obtained from the University of Venda Higher Degree Committee. Ethical clearance (no. SARDF/11/CRD/001) was obtained from the University of Venda Research Ethics Committee. Written permission to conduct the study in Mutale HBC organisations was obtained from the Limpopo Department of Health and Social Development, Vhembe District and Mutale Municipality.

Participants were given full information about the study procedures using the Tshivenda language, which they understood because it was their home language. Participants who were interested to participate in the study were given consent forms to sign, which some of them chose not to sign. Participants were informed about voluntary participation and their right to withdraw from the study at any time without the fear of being victimised. Confidentiality, privacy and anonymity were ensured throughout the study. Participants were informed not to write their names on the questionnaire; instead codes were used in order to ensure anonymity. Data collection took place in offices of HBC where no other persons were allowed when the questionnaires were administered in order to ensure privacy.

## Results

### Demographic characteristics

As shown in Tables 1–4, of 190 participants, 186 (97.8%) were women, and 172 (90.5%) were over the age of 35 years. The results showed that 24 (12.6%) participants had passed matric or grade 12. The majority of participants, 123 (64.7%), were staying with their partners, whilst 67 (35.3%) were single parents. The results further revealed that the distance travelled by voluntary home-based caregivers when visiting their clients to provide HBC ranged from 5 to more than 20 km per day as shown in Table 4.

**TABLE 1:** Participants’ gender and formal education (*N* = 190).

Variable	No formal education *n* (%)	Primary education *n* (%)	Secondary education without matric *n* (%)	Secondary education with matric *n* (%)	Tertiary education *n* (%)	*N* (%)
**Gender**						
Female	7 (3.7)	66 (34.7)	85 (44.7)	24 (12.6)	4 (2.6)	186 (97.9)
Male	-	-	-	4 (2.1)	-	4 (2.1)

**Total**	**7 (3.7)**	**66 (34.7)**	**85 (44.7)**	**24 (12.6)**	**4 (2.6)**	**190 (100.0)**

*Source*: Authors’ own work

**TABLE 2:** Participants’ ages.

Variable	Ages in years					

	18–25 *n* (%)	26–35 *n* (%)	36–45 *n* (%)	46–55 *n* (%)	56 and above *n* (%)	Total *n* (%)
**Gender**						
Female	1 (0.5)	17 (8.9)	126 (66.3)	39 (20.5)	3 (1.6)	186 (97.9)
Male	-	4 (2.1)	-	-	-	4 (2.1)

**Total**	**1 (0.5)**	**21 (11.05)**	**130 (68.4)**	**39 (20.5)**	**3 (1.6)**	**190 (100.0)**

*Source*: Authors’ own work

**TABLE 3:** Participants’ marital status (*N* = 190).

Gender	Married *n* (%)	Never married *n* (%)	Divorced *n* (%)	Widow/er *n* (%)	Total *n* (%)
Female	120 (63.1)	27 (14.2)	15 (7.9)	24 (12.6)	186 (97.9)
Male	3 (1.6)	1 (0.5)	-	-	4 (2.1)

**Total**	**123 (64.7)**	**28 (14.7)**	**15 (7.9)**	**24 (12.6)**	**190 (100.0)**

*Source*: Authors’ own work

**TABLE 4:** Distance travelled per day by participants when visiting clients.

Distance (in km)	Total *n* (%)
0–5	6 (3.2)
6–10	22 (11.6)
11–20	27 (14.2)
≥20	135 (71.0)

**Total**	**190 (100.0)**

*Source*: Authors’ own work

The demographic characteristics showed that the majority of voluntary home based caregivers’ ages was below 55 years. The results further revealed that none of the participants had any formal employment except HBC training.

### Impact of caregiving on community home-based caregivers

Voluntary home-based caregivers were asked to indicate how home-based caregiving impacted their lives during the last 12 months. Voluntary home-based caregivers were requested to select options that were related to their experiences, namely: never, sometimes or always as shown in Table 5.

**TABLE 5:** Self-reported impact of caregiving on participants (during the last 12 months, how often have you experienced any of the following?) (*n* = 190).

Impact of caregiving on caregivers	Never *n* (%)	Sometimes *n* (%)	Always *n* (%)
I feel depressed due to caregiving.	55 (29.0)	120 (63.0)	15 (8.0)
My social life have suffered because of caregiving.	99 (52.0)	76 (40.0)	12 (8.0)
I cannot do what I want because of caregiving.	95 (50.0)	63 (33.0)	32 (17.0)
I feel mentally exhausted as a result of caregiving.	62 (33.0)	109 (57.0)	19 (10.0)
I feel physically exhausted as a result of caregiving.	27 (14.1)	136 (72.0)	27 (14.0)
I feel worried about my future because I am not a registered worker.	10 (5.2)	79 (41.6)	101 (53.2)
I worry about getting infection from my client.	44 (23.2)	72 (37.8)	74 (39.0)
I use my stipend to help the person/s that I am caring for, for example buying bread.	77 (40.5)	86 (45.3)	27 (14.2)
I feel helpless when I cannot assist in the families I visit because of limited resources.	38 (20.0)	97 (51.0)	55 (29.0)
I feel like giving up this caregiving role.	111 (58.0)	70 (37.0)	9 (5.0)

*Source*: Authors’ own work

The results showed that 136 (72.0%) participants sometimes experienced physical exhaustion as a result of caregiving, whilst 15 (8.0%) always felt depressed. Even though the results showed that majority of participants 101 (53.2%) were always worried about their financial future because they were not registered as workers, the results showed that only 19 (10.0%) participants were mentally exhausted because of caregiving. The results further showed that majority 74 (39.0%) of participants were always worried about getting infection from their clients.

The results of this study shown in Table 5 indicate that caregiving has a negative financial impact on the lives of voluntary home-based caregivers. However, many home-based caregivers (111 [58%]) felt that they can never give up the caregiving role. Therefore, no matter how caregiving can impact negatively on the lives of home-based caregivers, they would continue to work as volunteers in HBC.

## Discussion

The results of this study showed that majority of voluntary home-based caregivers sometimes experienced mental exhaustion and felt depressed because of home-based caregiving. Similarly, the findings of a study conducted on primary family caregivers of patients with advanced cancer revealed that ‘caregivers reported greater depressive mood due to caregiving tasks’.^10^ Another study conducted by Yikilkan et al.^11^ on family caregivers revealed ‘depressive symptoms and severe depression amongst caregivers resulting from the high burden of caregiving’. ‘Voluntary home-based caregivers suffered emotionally when they find that the physical condition of the patient that they are supposed to take care of is very bad’.^8^

Various studies on HBC showed that ‘home-based caregiving has negative impact on the physical and emotional well-being of home-based caregivers’.^8,12^ Voluntary home-based caregivers find themselves performing many activities, which makes them ‘exhausted physically’.^13^ The results of the study revealed that voluntary home-based caregivers felt physically and mentally exhausted because of the caring activities. Voluntary home-based caregivers are involved in various activities when visiting patients such as cooking, washing linen and clothes, cleaning and carrying buckets of water in areas where there is no running tap water.^8,12,13^ These activities alone may result in physical exhaustion of the voluntary home-based caregiver.

The results of this study indicated that voluntary home-based caregivers were always worried about their future because they were not registered as workers and therefore felt emotionally stressed. Taking into consideration the profiles of voluntary home-based caregivers in this study who were unemployed, and were living in poverty, it was therefore relevant for voluntary home-based caregivers to be worried about proper employment with a salary. This finding is supported by Thabethe,^8^ who indicated that ‘voluntary home-based caregivers suffer from physical and emotional stress because they work long hours in home-based care providing free labour’. Voluntary home-based caregivers are taking some of the responsibilities of community health nurses who are insufficient; therefore, voluntary home-based caregivers are closing a gap created by the understaffed and underfunded public healthcare system by providing better care to people living with HIV and/or AIDS.^14^ Gilbert and Selikow^15^ in their study also found that ‘home-based care programmes are serving as substitute for formal health care system as the public health care systems are struggling under the strain of HIV and AIDS’.

The results of the study showed that majority of participants were worried about getting infection from their patients during the provision of care. Similarly, Akintola and Hangulu^16^ in their study on home-based caregivers of HIV and/or AIDS patients in Kwazulu Natal, South Africa, found that ‘voluntary home-based caregivers were exposed to the risk of infection with TB or HIV because of the shortage of protective devises such as disposable gloves’. In the same study ‘voluntary home-based caregivers reported to re-use disposable gloves or use empty bread plastic bags in the place of gloves’. ‘Lack of supplies such as home-based care kits was reported as a problem’ in home-based caregiving and can expose home-based caregivers to infection if there is no protective equipment.^17^ In their study on the perceived needs for support of voluntary home-based caregivers, Mashau et al.^18^ found that voluntary home-based caregivers ‘experienced shortage of protective equipment such as disposable hand gloves and aprons and could not always protect themselves when providing care to clients in their home settings’.

Despite the negative impact of caregiving on voluntary home-based caregivers, 111 (58.0%) participants in this study indicated that they have never felt like giving up the caregiving role. The results of a study conducted by Thabethe^8^ revealed that ‘voluntary home-based caregivers engage in community home-based care activities with the hope of acquiring work experience to secure formal job opportunities’. In this study, voluntary home-based caregivers revealed a lot of the negative impact resulting from caregiving; however, they still showed their commitment to caregiving in HBC.

### Limitations

Despite the sample size which was calculated proportionally from each HBC organisation, the sample size was small and the study was conducted in one municipality only.

## Conclusion

The results highlight the negative self-reported impact of caregiving on physical, mental and financial well-being of voluntary home-based caregivers. The results of this study highlight the need for support of voluntary home-based caregivers to promote the sustenance of HBC. Because majority of voluntary home-based caregivers were unemployed, they were worried about their future because they were not registered as workers. It seemed as if HBC was regarded as a form of employment by some voluntary home-based caregivers.

### Recommendations

Policies should be developed to address the issue of developing a body to regulate HBC. HBC policies should focus on the health of voluntary home-based caregivers. Collaborations with non-governmental organisations, government, local businesses and employers may assist in the development of income generation schemes for community home-based caregivers. Community stakeholders should be actively involved in the management and sustenance of their local HBC programme by applying for national and international funding for their HBC organisation. A programme to support voluntary home-based caregivers should be developed and implemented.

## References

[1] Smith KL, Soriano TA, Boal J Brief communication; national quality of care standards in home-based primary care. Annals Intern Med. 2007;146:188–192. http://dx.doi.org/10.7326/0003-4819-146-3-200702060-00008 10.7326/0003-4819-146-3-200702060-00008 17283350

[2] Fernandes R, Braun KL, Ozawa J, Compton M, Guzman C, Somogyi-Zalud E Home-based palliative care services for underserved populations. J Palliat Med. 2010;13(4):413–419. http://dx.doi.org/10.1089/jpm.2009.0216 20136521 10.1089/jpm.2009.0216

[3] Uys L, Cameron S Home-based HIV/AIDS care. South Africa: Oxford University Press; 2003.

[4] South African National Department of Health National guideline on home-base care/community-based care. Pretoria: Government Printers; 2001.

[5] World Health Organization (WHO) Community home-based care in resource-limited settings. A framework for action. Geneva, Switzerland: WHO; 2002.

[6] Lindsey E, Hirschfield M, Tlou S Home-based care in Botswana: Experiences of older women and young girls. Health Care Women Int. 2003;24(6):486–501. http://dx.doi.org/10.1080/07399330390199384 12851169 10.1080/07399330390199384

[7] South African National Department of Social Development Situational analysis and needs assessment of capacity among home and community based care service providers. Pretoria: Government Printers; 2006.

[8] Thabethe N Community home-based care – A cost effective model of care: Who benefits? AIDS Care. 2011;23(7):787–791. http://dx.doi.org/10.1080/09540121.2010.487086 21170764 10.1080/09540121.2010.487086

[9] Guilford JP, Frucher B Fundamental statistics in psychology and education. New York: McGraw-Hill; 1973.

[10] Govina O, Kronoulas G, Mystakidou K, Katsaragakis S, Vlachou E, Patiraki E Effects of patient and personal demographic, clinical and psychosocial characteristics on the burden of family members caring for patients with advanced cancer in Greece. Eur J Oncol Nurs. 2015;19(1):81–88. http://dx.doi.org/10.1016/j.ejon.2014.06.009 25442373 10.1016/j.ejon.2014.06.009

[11] Yikilkan H, Aypak C, Görpelioḡlu S Depression, anxiety and quality of life in caregivers of long-term home care patients. Arch Psychiatr Nurs. 2014;28:193–196. http://dx.doi.org/10.1016/j.apnu.2014.01.001 24856272 10.1016/j.apnu.2014.01.001

[12] Jack BA, Kirton J, Birakurataki J, Merriman A ‘A bridge to the hospice’: The impact of a community volunteer programme in Uganda. Palliat Med. 2011;25(7):706–715. http://dx.doi.org/10.1177/0269216310397566 21402659 10.1177/0269216310397566

[13] Orner P Psychosocial impacts on caregivers of people with AIDS. AIDS Care. 2006;18(3):236–240. http://dx.doi.org/10.1080/09540120500456565 16546784 10.1080/09540120500456565

[14] Rödlach A Home-based care for people living with AIDS in Zimbabwe: Voluntary caregivers’ motivations and concerns. Afr J AIDS Res. 2009;8(4):423–431. http://dx.doi.org/10.2989/AJAR.2009.8.4.6.1043 25875706 10.2989/AJAR.2009.8.4.6.1043

[15] Gilbert L, Selikow T ‘The epidemic in this country has the face of a woman’: Gender and HIV/AIDS in South Africa. Afr J AIDS Res. 2011;10(Suppl 1):325–334. http://dx.doi.org/10.2989/16085906.2011.637732 25865509 10.2989/16085906.2011.637732

[16] Akintola O, Hangulu L Infection control in home-based care for people living with HIV/AIDS/TB in South Africa: An exploratory study. Glob Public Health. 2014;9(4):382–393. http://dx.doi.org/10.1080/17441692.2014.895405 24697215 10.1080/17441692.2014.895405

[17] Moetlo GJ, Pengpid S, Peltzer K An evaluation of the implementation of integrated community home-based care services in Vhembe District, South Africa. Indian J Palliat Care. 2011;17(2):137–142. http://dx.doi.org/10.4103/0973-1075.84535 21976854 10.4103/0973-1075.84535 PMC3183603

[18] Mashau NS, Netshandama VO, Mudau MJ Voluntary home-based caregivers’ perceived needs for support: A study in the Mutale Municipality in South Africa. J Soc Sci. 2015;44(1):66–71.

